# The Effect of Dietary Intake and Nutritional Status on Anthropometric Development and Systemic Inflammation: An Observational Study

**DOI:** 10.3390/ijerph18115635

**Published:** 2021-05-25

**Authors:** Roxana Maria Martin-Hadmaș, Ștefan Adrian Martin, Adela Romonți, Cristina Oana Mărginean

**Affiliations:** 1 Department of Community Nutrition and Food Safety, “George Emil Palade” University of Medicine Pharmacy Science and Technology of Târgu Mureș, 540139 Târgu Mureș, Romania; roxana.hadmas@umfst.ro (R.M.M.-H.); adela.romonti@umfst.ro (A.R.); 2 Center for Advanced Medical and Pharmaceutical Research, Department of Physiology, “George Emil Palade” University of Medicine Pharmacy Science and Technology of Târgu Mureș, 540139 Târgu Mureș, Romania; 3 Department of Pediatrics I, “George Emil Palade” University of Medicine Pharmacy Science and Technology of Târgu Mureș, 540139 Târgu Mureș, Romania; oana.marginean@umfst.ro

**Keywords:** interleukin, cytokine, skin folds, respiratory coefficient, inflammation

## Abstract

(1) Background: Daily caloric intake should aim to reduce the risk of obesity or poor anthropometric development. Our study objective was to analyze the association between food consumption, inflammatory status and anthropometric development; (2) Methods: We performed a prospective observational analytical research during September 2020 and April 2021 on a group of 160 healthy subjects, aged between 6 and 12 years old, by analyzing food ingestion, the basal metabolic rate, anthropometric development and the inflammatory status; (3) Results: IL-6 was significantly correlated to the sum of skinfolds, along with both serum proteins and triglycerides. The skin folds were significantly correlated with the caloric intake and with total fat intake, next to saturated and trans fats. Unlike the skin folds, the body weight was significantly correlated with the caloric intake along with some vitamins, such as Vitamin A and Vitamin B12. Inactive mass increased with excessive folic acid, Vitamin E, Vitamin K and saturated fat intake; (4) Conclusions: The inflammatory status was influenced by the ingestion of micronutrients, total serum lipids and proteins. The anthropometric development was associated with the ingestion of carbohydrates, energy balance and energy intake. We can conclude that daily menu and nutrition imbalances can influence both the risk of obesity and the inflammatory status.

## 1. Introduction

Dietary imbalance causes changes either an excess or deficit in body weight, depending on the nutritional needs and the caloric intake. As known, physically inactive individuals that have a positive caloric balance may report a higher risk of obesity. On the other hand, a negative energy balance due to lack of food intake may increase the risk of malnutrition. In both cases, the consequence is improper anthropometric development, which means that each condition must be treated equally.

Daily caloric intake should aim to reduce the risk of obesity or poor anthropometric development [1]. Due to food intake, various functional and anthropometric changes occur in children, which can furthermore affect the age-specific development process [2,3]. However, the consequences of poor anthropometric development during childhood period are much more accurately observed in adults [4,5,6], who further develop cardiovascular events, metabolic along with systemic and pulmonary dysfunctions [6,7,8]. All these clinical manifestations were previously seen by studying the cytokines and, particularly, interleukins [9].

Changes in inflammatory status are mainly seen in adults [10], while limited data are published on young individuals [11]. As seen in several research papers, changes in body weight failed to increase the inflammatory status [12,13]. Overall, the main changes in inflammatory status are related to various clinical outcomes. The most common are diabetes, insulin resistance, dyslipidemia, hypertension with possible cardio-circulatory manifestations [14,15,16].

Few papers approached topics related to food intake and systemic changes [17,18,19]. The only exceptions are the papers that studied caloric intake and anthropometric changes [20,21]. This is why it becomes clear that the theoretical and practical approach of anthropometric analysis is usually related to daily energy intake and less on daily nutritional intake.

Following the worldwide obesity rate, various intervention and diagnostic procedures are in continuous development [21,22]. Numerous research papers studied the nutrition value and the caloric intake while measuring the main changes in anthropometrics or the association between food groups and health risk [23,24,25]. Therefore, we know that whole grains unlike refined grains induce less important changes in body weight, that vegetables and legumes reduce the risk of obesity or that an improvement in the body weight is usually seen if fruits are consumed within the limit of three hundred grams per day [26]. For all that, most of the food products increase the risk of obesity. The only condition is the quantity in relationship with daily energy requirements and the amount of nutrients obtained through food intake. Nutrient intake may induce changes in inflammatory status, specific in IL-6 and IL-8 levels, under main changes in the body mass.

Our study objective was to analyze the association between food consumption, inflammatory status and anthropometric development. The hypothesis is that daily energy intake together with several nutrients can influence the risk of obesity and the inflammatory ratio through IL-6 and IL-8.

## 2. Materials and Methods

### 2.1. Study Design

We performed a prospective observational analytical research during eight months period, over September 2020 and April 2021. All procedures, tests and measurements were performed in the Advanced Medical and Pharmaceutical Research Center (CCAMF) of ”George Emil Palade” University of Medicine, Pharmacy, Science and Technology of Târgu Mureș, Romania, on a group of 160 participants. No more than 18 participants were excluded due to a pre-existing chronic inflammatory status.

Our research protocol was accepted (No. 259/14.11.2018) by the Ethical Committee of the ”George Emil Palade ” University of Medicine, Pharmacy, Science and Technology of Târgu Mureș, Romania and at the same time the protocol complied with the mentions regarding human experimentation and research protocols as required in the Declaration of Helsinki. Prior to research, all means of research were explained to the children and their parents/ caregivers. If the research measures were understood and accepted at the same time, the parents/caregivers signed the informed consent and the acceptance for their children to participate in the research.

### 2.2. Study Participants

The following inclusion criteria were applied: children aged between 6 and 12 years old with no food allergies, acute or chronic pathologies. If any of these criteria were not met, the participants were excluded from the research.

### 2.3. Test Applied

**Figure 1:**
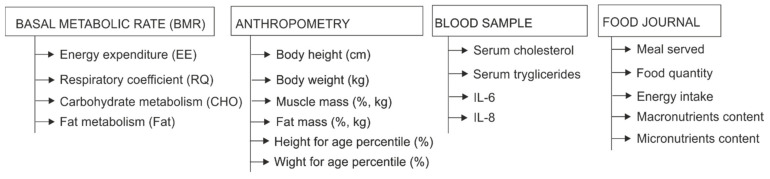
Study sample research procedures.

During one single visit, the participants went through the basal metabolic rate (BMR) measurement, next to the anthropometric measurements and the blood sampling. The food intake was analyzed by using a food diary, completed by the parent/ caregiver over 7 days period, as illustrated in Figure 1.

### 2.4. Basal Metabolic Rate (BMR)

We used the indirect calorimetry method to measure the BMR. Each measurement took place after a 12-h fasting period by using the Cortex Metalyzer 3B equipment (Cortex Medical, Leipzig, Germany) the MetaSoft 3 software (Cortex Medical, Leipzig, Germany) and the facemask. Study participants were not allowed to exercise for 48 h before testing, and were recommended a normal, balanced diet.

The equipment was calibrated before each test with known O2 and CO2 concentrations, while the participants were adapting to the testing environment. In the beginning of the test, we measured heart rate (HR, bpm), the systolic (SBP, mmHg) and diastolic blood pressures (DBP, mmHg) by using General Electric HEALTCARE B20 device (General Electric, Boston, MA, USA). From the supine position, the energy expenditure was measured as kilocalories (kcal) per minute/hours/day. The respiratory coefficient (RQ) was measured next to oxygen consumption (VO2, L/min) and carbon dioxide production (VCO2, L/min). By using these parameters, we appreciated the carbohydrate metabolism (CHO, grams per day, %) next to the fat metabolism (Fat, grams per day, %). Additional information was provided through the standard protocol and the MetaSoft 3 use: body mass index (BMI) and resting energy requirement (kcal/day) which was calculated automatically by applying the Harris Benedict equation [27].

### 2.5. Anthropometric Measurements

The participants underwent anthropometric measurements during one single visit. In the presence of the parent/caregiver, the participants remained in minimal clothing during all anthropometric measurements. The body weight and the body height were the common measurements. Each measurement took place in similar conditions as the BMR test, after a 12-h fasting period by using the ADE, GmbH M30404-01 calibrated thaliometer (ADE Germany GmbH, Hamburg, Germany).

We used the HARPENDEN Professional Skinfold equipment (Baty International Ltd, Burgess Hill, United Kingdom) to measure the following skinfolds: the biceps, the triceps, the subscapular, the suprailiac, the abdominal, the thigh and the leg skin fold. Allover, the age, the gender, the height and the weight next to the biceps, the triceps, the subscapular and the suprailiac skinfold values were used in the Durnin and Womersley formula [28] to determine the body mass: muscle mass, fat mass, illustrated as kilograms (kg) and percentage of the total body weight (% of total body mass).

All anthropometric measurements took place in three successive repetitions at 20-s intervals. For each measurement, we calculated the average value that was later used in statistics. For each participant, we extrapolated the height for age (%) and the weight for age (%) percentiles. The normal range was listed between 5 and 95%. Under 5%, the participants were underweight and over 95%, the participants were considered overweight [29].

### 2.6. Blood Samples

Following similar fasting conditions, 15 mL of venous blood were collected from each participant. After each collection, the blood samples were kept for 15 min at room temperature, were centrifuged at 80 rpm and sampled in 12 µL micro tubes. In the end, the blood samples were frozen at −80^◦^C and stored until analyzed. All samples were analyzed on one occasion. The Cobas Integra 400 Plus (F. Hoffmann-La Roche AG, Baselz, Switzerland) was used to determine the level of cholesterol (80–200 mg/dL), creatinine (<8 years old: 0.40–0.60 mg/dL; 8–10 years old: 0.39–0.73 mg/dL), triglycerides (50–150 mg/dL) and total proteins (66–87 g/L). We determined both IL-6 (<3.8 pg/mL) and IL-8 (<15 pg/mL) serum levels. Each parameter was measured from the sample by using the DYNEX DSX AUTOMATED ELISA SYSTEM (Dynex® Technologies, Inc., Chantilly, VA, USA) based on an immuno-enzymatic assay.

The reagents were brought to room temperature. During the first stage, the samples, standards and controls were added to the wells, which were lined with a monoclonal antibody for IL-6 and IL-8. After the incubation period, the IL-6 and IL-8 present in the sample, standard or control unrelated to the fixed antibody was removed by washing with a wash buffer. After the washing process, a solution containing a monoclonal antibody to IL-6 labeled with an enzyme-biotin was added. We further added the streptavidin-HRP conjugate. The analyzer read the absorbance of each well spectrophotometrically. The IL-6 and IL-8 concentrations were determined after reading the absorbance for each well and after the interpolation on the calibration curve. The cytokine concentration was directly proportional to the color intensity of the well.

### 2.7. Daily Food Intake

We used a seven-day (n = 7) food journal to get data over daily food intake. The journal was made available to the participants by using the google form platform (Mountain View, CA, United States). Completion time was estimated at 10 minutes. Each journal had three sections. The first section was to identify the participant. Section 2 was used to detail food intake and Section 3 was used to detail fluid intake.

Every day we received information regarding food intake, time, type of food and food quantity. The food quantity was measured by using calibrated scales, which were made available to them during the research. Fluid intake was measured as well by using the third section of the questionnaire. The intake was estimated by reporting the amount consumed daily (liters per day).

We used USDA’s Food Composition Databases [30], a property of the United States’ Department of Agriculture (2019), to analyze daily food intake. Thus, we conducted a quantitative and qualitative analysis, by using the following information for each food product: caloric value (kcal), carbohydrate (g), protein (g) and lipids (g) content, along with cholesterol (mg), saturated fats (g), monounsaturated fats (g), polyunsaturated fats (g), trans fats (g), sugars (g) and fibers (g). Further, Iron (mg), Calcium (mg), Magnesium (mg), Phosphorus (mg), Potassium (mg), Sodium (mg), Zinc (mg), Selenium (mg), Folic Acid (µg), Vitamin A (µg), Vitamin C (mg), Vitamin B12 (mg), Vitamin B1 (mg), Vitamin B6 (mg), Vitamin K (µg) and Vitamin E (mg) were measured as well.

### 2.8. Statistical Evaluation

The statistical evaluation was carried out with the GraphPad Prism 6.0 software (Graph Pad Software, San Diego, CA, USA), with a level of significance set at α = 0.05. The tests used for the inferential assessment were: Mann–Whitney test for the differences and the Spearman r test for assessing the relation between two analyzed parameters. The data were presented by using descriptive data as the median value, the minimum-maximum values and the variation coefficient (CV). Due to food intake variability, the average values were not used in this study.

## 3. Results

The research results are illustrated by applying a systematic passage through each relevant subject regarding food intake, caloric need and related changes in body mass.

### 3.1. Demographic Analysis

All participants passed the anthropometric measurement stage. The weight and the height were measured to determine the body mass and the anthropometric development stage according to age.

The median age in the study group was 10.3 years old (6 to 12 years old), CV = 19.67%. Following the anthropometry measurements, the median body weight was between 22.7 and 75.5 kg with a median value of 46.1 kg and 26.07% CV. The body weight for age was 57%, whereas the height for age reached 67%, both with maximum values near 100%. Furthermore, the BMI was 18.5, while the fat mass ratio was between 6.6 and 39.3 with 16.7% median value.

### 3.2. Basal Metabolic Rate—Energy Expenditure

Based on the BMR measurement, oxygen consumption was 0.24 L/min, unlike 0.21 L/min VCO2. The basal metabolic rate reached 1676 kcal/day, while the resting theoretical energy needs were 1376 kcal/day. The difference between the two numerical values was 17.9% (*p* = 0.0001, r = 0.653).

By measuring the O2 consumption and the CO2 production, RQ was 0.86, CV = 18.68%. Following the BMR measurement, the carbohydrate metabolism was 221 grams per day (CV = 55.79%) compared to the fat metabolism, which reached 74 grams per day (CV = 38.9%), as illustrated in Table 1.

The carbohydrate metabolism was equivalent to 906.1 kcal median value and 54.06% of the daily energy requirements, unlike fat metabolism, which was equivalent to 688.2 kcal, and 41.06% of the daily energy needs. We assume that the remaining energy is allocated to protein, meaning no more than 5 to 10% and 83.8 to 167.6 kcal/day equivalent to 20.31 to 40.87 grams of protein per day. 

### 3.3. Nutritional Intake

The median protein intake was 79.2 g/day, followed by 72.53 g/day fat and 207.6 g/day carbohydrate, as further detailed in Table 2.

Compared to the basal energy requirements, there are important differences in the daily intake. Carbohydrate needs (221 g/day) are significantly different to carbohydrate intake (207.6 g/day), as seen through *p* = 0.0383, Mann–Whitney U = 8104, despite minimal quantitative differences in median values. We did not obtain a similar result regarding fat intake (*p* > 0.05). However, during the BMR measurement the lipid requirements were 74 g/day, unlike 72.53 g daily intake (*p* > 0.05). Similar results were found in daily micronutrient intake (Table 3).

### 3.4. Daily Food Intake: Calories, Nutrients and Influence over the Blood Samples

Serum IL-6 was between 0.05 and 5.98 with a median reach of 1.41 pg/mL. IL-8 was between 0.72 and 38.9 with a median value of 7.09 pg/mL. The cholesterol median value was 144.7 mg/dL, with maximum values reaching up to 245.3 mg/dl, whereas triglycerides reached 49.46 mg/dL median value, with maximum values of 150 mg/dL. All measures were correlated to energy and macronutrient intake, as further illustrated in Table 4.

Overall, food intake influenced the inflammatory status under both changes in body weight and body mass. As such, IL-6 value was significantly correlated with both polyunsaturated fats (*p* = 0.0045, r = 0.27) and daily fiber intake (*p* = 0.0005, r = −0.33), while daily sugar intake was significantly correlated with changes in serum parameters (*p* = 0.0186, r = 0.226). Further, Vitamin A (*p* = 0.0489, r = −0.19), Vitamin C (*p* = 0.0181, r = −0.2272) and Vitamin B1 (*p* = 0.0064, r = −0.2609) where all correlated to IL-6 concentration. With one exception: calcium intake (*p* = 0.0389, r = −0.199), daily food intake failed to correlate with IL-8 (*p* > 0.05), as further detailed in Table 5.

As cholesterol was higher, the serum concentration of total proteins (*p* = 0.0009, r = 0.272) and body weight (*p* = 0.0192, r = 0.200) were as well higher. Yet, IL-6 was significantly correlated to the sum of the skin folds (*p* = 0.0289, r = 0.187) along with both serum proteins (*p* = 0.0192, r = 0.2006) and triglycerides (*p* = 0.015, r = 0.208). As expected, the skin folds were significantly correlated to the body weight (*p* = 0.0203, r = 0.188) result which confirms the existing relationship between food intake, body mass and body weight.

The skin folds were significantly correlated with the total caloric intake and with total fat intake (*p* = 0.0015, r 0.297), next to saturated (*p* = 0.0005, r = −0.3221) and trans fats (*p* = 0.0467, r = 0.188). Carbohydrate intake (*p* = 0.0259, r = 0.210) was correlated with the body weight and with sugar intake (*p* = 0.0173, r = 0.224). Unlike the skin folds, the body weight was significantly correlated with the caloric intake (*p* = 0.035, r = 0.165) along with some vitamins, such as: Vitamin A (*p* = 0.0106, r = 0.234) and Vitamin B12 (*p* = 0.0324, r = 0.197). Inactive mass increased with excessive folic Acid (*p* = 0.0101, r = −0.235), Vitamin E (*p* = 0.0007, r = −0.306), Vitamin K (*p* = 0.0124, r = −0.229) and saturated fats (*p* = 0.0287, r = 0.201).

## 4. Discussion

### 4.1. Basal Metabolic Rate vs. Daily Food Intake

In many situations, the information regarding the energy demand is obtained by using equations. Through these, we notice that the differences between direct and indirect methods do not differ most of the time by more than 5–10% [31,32], but there are exceptions that can reach up to 40% differences [33]. In our case, the basal metabolism rate reached a median of 1676 kcal versus 1376 kcal. These indicate a difference of almost 18%, with higher measured values.

As in other papers [34,35], active muscle tissue increased the basal metabolic rate. However, the decrease in energy requirements was not necessarily observed in our study group due to obesity but rather due to the body weight. This result is opposite to Friedman IM et al. [36]. Friedman IM et al. reported lower basal metabolic rate and changes in body mass; this is why these differences are more related to body mass distribution, physical activity level and gender.

Food intake has both an energy and a nutritional component, starting from the role and the importance of each macronutrient. Even if there are important metabolic differences between carbohydrates, proteins and lipids, we cannot question the presence of one, the lack of another and thus the body weight control [36]. In our paper, 906.1 kcal were attributed to carbohydrate metabolism, equivalent to 54.06% of the energy demand. This value covers the participant’s needs. However, we had cases in which the carbohydrate intake decreased. By dropping carbohydrate intake, the energy requirements increased. Under these circumstances, Veldhorst MAB et al. [37] reported that gluconeogenesis increases energy requirements by changes in protein turnover. On the other hand, simple carbohydrates increase the risk of positive changes in fat mass, as detailed by Sartorius K et al. [38] who published similar changes with various clinical manifestations, such as the metabolic syndrome, type II diabetes and various forms of vascular damage.

As against carbohydrates and fats, protein intake increases satiety. This is also the reason why the energy intake decreases. According to Carbone JW et al. [39], an energy restricted, but high protein intake can increase the active tissue and reduce the adipose tissue, whereas Martens EA et al. [40] state that high protein intake can maintain energy expenditure, as against lower protein intake. Somewhat similar, protein intake was correlated with body mass in our paper. According to Westerterp-Plantenga et al. [41] by increasing the protein intake, one can maintain a balanced body weight.

Dietary fats can have important roles in several metabolic disorders, while affecting insulin resistance in normal weight individuals [42]. In our study, fat intake reached 72 grams per day, equivalent to 673 kcal and almost 41% of the daily energy intake. However, the fat intake failed to corelate with changes in body weight, similar to Beulen Y et al. [43]. In his paper, fat intake increased energy expenditure and failed to increase the body weight over a short–medium period of time. Further, in several studies’ fat intake brought minimal changes in body fat mass [44,45]. This is also the reason why on many occasions’ fat intake does not significantly influence body mass, as we have seen in our research.

### 4.2. Food Intake, Anthropometric Differences and Changes in Blood Samples

Daily energy intake influences anthropometric development and various serum biochemical parameters. As seen in our results, micronutrients can influence the inflammatory status, starting from changes in body weight usually related to macronutrients and energy intake.

In the current research, the energy intake failed to correlate to serum cholesterol, triglycerides next to both IL-6 and IL-8. Furthermore, the macronutrients failed to correlate with serum parameters. The only exceptions are the carbohydrates next to the proteins, which were statistically significantly correlated with total serum proteins, unlike Praveen S et al. [46]. Therefore, a higher carbohydrate intake was related to lower protein consumption and lower body weight, similar to Bopp MJ et al. [47], unlike Nabuco HCG [48], which obtained a drop in serum proteins based on high carbohydrate but normal protein intake. These comparisons can also be described by the glycogenosis process, where the protein turnover is altered. As to fat intake, the resulting cholesterol by using trans-fatty acids were significantly correlated to both serum cholesterol and triglycerides, without an influence on IL-6 and IL-8 concentrations, similar to Koga N et al. [49].

Inflammatory status will change with body mass, but not with daily food intake. Yet, saturated fats failed to correlate, whereas polyunsaturated fats were negatively correlated, which shows us that quality fats can decrease the inflammatory status. However, during a short-term period, fat intake failed to influence the inflammatory status as described by Telle-Hansen VH et al. [50] in a review paper. The carbohydrates increase the risk of obesity, while normal protein and fat intake can maintain the energy expenditure and therefore the health condition. Yet, sugars, trans-fatty acids, cholesterol and unsaturated fats can easily increase the cholesterol, triglycerides and subsequently, through the energetic substrate, the body weight with potential on the inflammatory status as seen in our paper.

Unexpected, micronutrients influence the serum changes discussed earlier. Most likely, the main changes are secondary to food intake, which can provide a smaller or larger amount of each micronutrient. Therefore, the daily calcium intake was significantly correlated to both IL-8 and serum proteins. An increased intake of calcium limited IL-8 concentration whereas being related to serum proteins. However, according to Hendy GN et al. [51] the cytokines contribute to altered calcium homeostasis, which further is related to daily intake. Magnesium was correlated to IL-6 and serum proteins, reflecting the fact that the higher the nutritional intake, the lower the risk of inflammatory status. As such Moslehi N et al. [52] studied the relationships between serum magnesium and the inflammatory markers, without reaching a significantly attenuate inflammatory state following 8 weeks period. Similar results are seen for vitamin A, B, B12, B1, B6, which were all correlated negatively with IL-6, IL-8 and serum lipid components. In several research papers [53,54,55,56,57], both vitamins and minerals were used to attenuate the inflammatory state. Yet, few of them managed to reduce the inflammatory status by using different micronutrients in various serving quantities. Many of them failed to demonstrate that micronutrients could positively influence the inflammatory status. However, based on the body weight changes, we can see that the daily intake can be related to the state.

By implementing the study protocol, we managed to analyze the anthropometric development of young individuals aged between 6 and 12 years old. The anthropometric study and its conclusions have a different connotation by its relationship with the daily food intake and the basal metabolic rate. Based on our study outcome, one can observe that there are limited statistical relationships between the macronutrient intake and body weight. A series of more important relationships were rather seen between caloric intake and body mass. We measured changes in body mass under fat intake, namely due to a higher saturated fat. The result was similar if significant amounts of simple carbohydrates were consumed, which limited the intake of various micronutrients. Overall, replacing processed foods with high nutrients products reduced the risk of dyslipidemia and the risk of changes in both IL-6 and IL-8 by less important changes in body mass.

Nevertheless, we further mention a number of limitations of the current research. The most important limitation is the study sample. This sample allows us to observe functional changes, while an exact conclusion is formulated by studying a larger sample. However, this research proposed a punctual analysis of energy metabolism, food intake and blood sample parameters. Among the blood tests, future research should include IL-4, IL-10 and IL-13, which we did not use, to draw conclusions that are more accurate on a larger group of participants. It also becomes important to sample the study group, based on the anthropometric data.

## 5. Conclusions

Each macronutrient has an important role in anthropometric development and health. In our study, the inflammatory status was influenced by the ingestion of micronutrients, total serum lipids and serum total protein unlike macronutrients. The anthropometric development was associated with the ingestion of carbohydrates, energy balance and energy intake. Specifically, there were no positive differences in carbohydrate intake by increasing sugar consumption compared to the energy and the nutritional needs. Furthermore, the intake of foods containing protein and lipids favored an increased cholesterol and serum triglycerides values, with positive changes in body weight, body mass and IL-6, but without significant changes in IL-8.

We can conclude that daily menu and nutrition imbalances can influence both the risk of obesity, which can, therefore, increase the inflammatory status and the risk of several nutritional and anthropometric health related issues, which are usually seen in both adolescents and adult groups with changes in body weight.

## Figures and Tables

**Table 1:** Carbohydrate, Fat and Protein metabolism (kcal) as measured and compared to the basal metabolic rate (BMR: 1676 kcal).

Macronutrient	Median (Min to Max)	% of the Median Energy Need
Carbohydrates (g/day)	906.1 (709.3 to 1143.9 kcal)	54.06 (42.32 to 68.25 kcal)
Fats (g/day)	688.2 (530.1 to 855.6 kcal)	41.06 (31.62 to 51.05%)

Legend: Min—minimum; Max—maximum; %—percentage; g/day—grams per day.

**Table 2:** Descriptive analysis regarding macronutrients intake.

Macronutrient		Median (Min to Max)	CV, %
Carbohydrates (g/day)		207.6 (100 to 393 )	29.37
	Sugars (g/day)	42.32 (10.5 to 110.9)	40.74
	Fibers (g/day)	25.51 (10.24 to 110.9)	30.41
Proteins (g/day)		79.2 (28.2 to 227.9)	36.63
Fats (g/day)		72.53 (16.55 to 164.3)	39.80
	Saturated fats (g/day)	735.4 (4.03 to 3966)	12.94
	Monounsaturated fats (g/day)	9.44 (0.70 to 42.5)	73.76
	Polyunsaturated fats (g/day)	4.03 (0.73 to 31.01)	93.85
	Trans fats (g/day)	0.08 (0 to 110.9)	134.72
	Cholesterol (g/day)	237.2 (14 to 818.8)	60.14

Legend: Min—minimum; Max—maximum; CV—Coefficient of variation; %—percentage; g/day—grams per day.

**Table 3:** Micronutrient’s intake in the study group.

Macronutrient		Median (Min to Max)	CV, %
Iron (mg/day)		15.2 (5.68 to 26.27)	32.52
Calcium (mg/day)		14.06 (90.9 to 3530)	50.69
Magnesium (mg/day)		140.1 (56.7 to 313.1)	43.53
Phosphorus (mg/day)		641.1 (141.7 to 2094)	48.59
Potassium (mg/day)		1713 (6936.3 to 3766)	38.79
Sodium (mg/day)		2868 (672.3 to 4493)	31.72
Zinc (mg/day)		8.25 (0.195 to 240.7)	109.67
Selenium (mg/day)		8.60 (0 to 78.6)	109.66
Vitamin	A (µg/day)	677.2 (83.52 to 29,171)	207.14
	C (mg/day)	28.87 (0.6 to 12.24)	86.37
	B12 (µg/day)	1.21 (0 to 12.24)	105.69
	B1 (mg/day)	0.93 (0.149 to 1.96)	46.71
	B6 (mg/day)	0.98 (0.33 to 3.00)	49.96
	K (µg/day)	16.95 (0 to 489.5)	183
	E (mg/day)	1.22 (0.118 to 8.07)	94.65
	Folic Acid (µg/day)	7.2 (0 to 386.6)	212.44

Min—minimum; Max—maximum; CV—Coefficient of variation; %—percentage; g/day—grams per day; mg/day—milligrams per day; µg/day—micrograms per day.

**Table 4:** Univariate analysis between energy intake, next to macronutrient intake and blood sample results.

Macronutrient Intakes (Median, Min to Max)	Laboratory Parameters (Median, Min to Max)									
	IL-6 (pg/mL) (1.41, 0.05 to 5.98)		IL-8 (pg/mL) (7.09, 0.72 to 38.9)		Cholesterol (mg/dL) (144.7, 91.33 to 245.3)		Triglycerides mg/dL) (49.46, 25.67 to 152.4)		Total Proteins (g/L) (69.13, 59.71 to 76.41)	
	*p*	r	*p*	r	*p*	r	*p*	r	*p*	r
Energy intake (kcal/day): 1854, 773.4 to 3606	0.446	−0.074	0.560	0.056	0.822	108	0.749	0.031	0.0327	0.205
Carbohydrate intake (g/day): 207.6, 100.3 to 393	0.114	−0.152	0.066	0.177	0.400	0.081	0.787	−0.026	0.006	−0.262
Fat intake (g/day): 72.53, 16.55 to 164.3	0.598	0.051	0.802	0.024	0.472	−0.069	0.633	0.046	0.1617	−0.135
Protein intake (g/day): 79.2, 28.2 to 227.9	0.847	0.018	0.862	−0.016	0.252	−0.111	0.333	−0.093	0.066	−0.177
Cholesterol (mg/day): 237.2, 14 to 818.8	0.179	0.130	0.321	−0.096	0.046	0.635	0.034	0.161	0.889	−0.0135
Saturated fat (g/day): 735.4, 4.03 to 3966	0.330	−0.094	0.918	−0.010	0.881	−0.014	0.069	−0.175	0.196	−0.125
Monounsaturated fats(g/day): 9.44, 0.70 to 42.5	0.179	−0.130	0.466	−0.070	0.191	−0.126	0.144	0.141	0.017	−0.227
Polyunsaturated fats (g/day): 4.03, 0.73 to 31.01	0.004	−0.271	0.613	−0.049	0.884	−0.014	0.468	0.070	0.028	−0.215
Trans fats (g/day): 0.08, 0 to 110.9	0.294	−0.101	0.202	−0.123	0.002	0.223	0.045	0.192	0.002	−0.286
Sugar (g/day): 42.32, 10.5 to 110.9	0.018	0.226	0.720	0.034	0.949	−0.006	0.048	0.190	0.449	−0.073
Fibers (g/day): 25.51, 10.24 to 110.9	0.000	−0.330	0.300	0.100	0.9009	0.012	0.039	−0.198	0.001	−0.297

Legend: Min—minimum; Max—maximum; g/day—grams per day; pg/mL—Picograms per milliliter; mg/dL—milligrams per deciliter; *p*—probability level; r—Pearson product-moment correlation coefficient.

**Table 5:** Univariate analysis between micronutrient intake and blood sample results.

Micronutrient Intakes (Median, Min to Max)	Laboratory Parameters (Median, Min to Max)									
	IL-6 (pg/mL) (1.41, 0.05 to 5.98)		IL-8 (pg/mL) (7.09, 0.72 to 38.9)		Cholesterol (mg/dL) (144.7, 91.33 to 245.3)		Triglycerides mg/dL) (49.46, 25.67 to 152.4)		Total Proteins (g/L) (69.13, 59.71 to 76.41)	
	*p*	r	*p*	r	*p*	r	*p*	r	*p*	r
Iron (mg/day): 15.2, 5.68 to 26.27	0.179	−0.130	0.213	0.120	0.838	0.019	0.062	0.179	0.154	−0.137
Calcium (mg/day): 14.06, 90.9 to 3530	0.285	−0.103	0.038	−0.199	0.092	−0.162	0.482	0.068	0.0001	−0.356
Magnesium (mg/day): 140.1, 56.7 to 313.1	0.047	−0.191	0.521	0.062	0.167	−0.133	0.192	0.126	0.040	−0.197
Phosphorus (mg/day): 641.1, 141.7 to 2094	0.335	−0.093	0.861	−0.017	0.163	−0.135	0.173	0.131	0.015	−0.232
Potassium (mg/day): 1713, 6936.3 to 3766	0.259	−0.109	0.395	0.082	0.018	−0.226	0.386	0.084	0.030	−0.208
Sodium (mg/day): 2868, 672.3 to 4493	0.384	−0.084	0.854	0.017	0.604	0.050	0.890	−0.013	0.0001	−0.366
Zinc (mg/day): 8.25, 0.195 to 240.7	0.582	0.053	0.067	0.176	0.5625	−0.056	0.005	0.265	0.493	−0.066
Selenium (mg/day): 8.60, 0 to 78.6	0.358	0.089	0.250	0.111	0.286	−0.103	0.371	0.086	0.158	−0.136
Vitamin A (µg/day): 677.2, 83.52 to 29,171	0.048	−0.19	0.984	0.001	0.012	−0.239	0.498	0.065	0.025	−0.215
Vitamin C (mg/day): 28.87, 0.6 to 12.24	0.018	−0.227	0.489	0.067	0.959	0.004	0.491	−0.066	0.051	−0.187
Vitamin B_12_ (µg/day): 1.21, 0 to 12.24	0.345	0.091	0.478	0.068	0.033	−0.205	0.021	0.221	0.536	−0.060
Vitamin B1 (mg/day): 0.93, 0.149 to 1.96	0.006	−0.260	0.056	−0.184	0.1844	−0.128	0.1647	0.134	0.002	−0.293
Vitamin B6 (mg/day): 0.98, 0.33 to 3.00	0.091	−0.163	0.195	0.125	0.301	−0.100	0.787	0.026	0.031	−0.206
Vitamin K (µg/day): 16.95, 0 to 489.5	0.0001	−0.404	0.134	−0.145	0.001	−0.303	0.2214	0.118	0.006	−0.261
Vitamin E (mg/day): 1.22, 0.118 to 8.07	0.002	−0.293	0.812	0.023	0.014	−0.234	0.1861	0.128	0.0001	−0.339
Folic Acid (µg/day): 7.2, 0 to 386.6	0.037	−0.200	0.359	0.088	0.574	−0.054	0.152	−0.138	0.066	−0.177

Min—minimum; Max—maximum; g/day—grams per day; pg/mL—Picograms per milliliter; mg/dL—milligrams per deciliter; mg/day—miligrams per day; µg/day—micrograms per day; *p*—probability level; r—Pearson product-moment correlation coefficient.
